# Expression of Truncated PITX3 in the Developing Lens Leads to Microphthalmia and Aphakia in Mice

**DOI:** 10.1371/journal.pone.0111432

**Published:** 2014-10-27

**Authors:** Kenta Wada, Yoshibumi Matsushima, Tomoki Tada, Sayaka Hasegawa, Yo Obara, Yasuhiro Yoshizawa, Gou Takahashi, Hiroshi Hiai, Midori Shimanuki, Sari Suzuki, Junichi Saitou, Naoki Yamamoto, Masumi Ichikawa, Kei Watanabe, Yoshiaki Kikkawa

**Affiliations:** 1 Department of Bioproduction, Tokyo University of Agriculture, Abashiri, Japan; 2 Mammalian Genetics Project, Tokyo Metropolitan Institute of Medical Science, Tokyo, Japan; 3 Research Institute for Clinical Oncology, Saitama Cancer Center, Saitama, Japan; 4 Graduate School of Life and Environmental Sciences, University of Tsukuba, Tsukuba, Japan; 5 Medical Innovation Center, Graduate School of Medicine Kyoto University, Kyoto, Japan; 6 Basic Research Center, Tokyo Metropolitan Institute of Medical Science, Tokyo, Japan; 7 Institute of Joint Research, Fujita Health University, Toyoake, Japan; University of Delaware, United States of America

## Abstract

Microphthalmia is a severe ocular disorder, and this condition is typically caused by mutations in transcription factors that are involved in eye development. Mice carrying mutations in these transcription factors would be useful tools for defining the mechanisms underlying developmental eye disorders. We discovered a new spontaneous recessive microphthalmos mouse mutant in the Japanese wild-derived inbred strain KOR1/Stm. The homozygous mutant mice were histologically characterized as microphthalmic by the absence of crystallin in the lens, a condition referred to as aphakia. By positional cloning, we identified the nonsense mutation c.444C>A outside the genomic region that encodes the homeodomain of the paired-like homeodomain transcription factor 3 gene (*Pitx3*) as the mutation responsible for the microphthalmia and aphakia. We examined *Pitx3* mRNA expression of mutant mice during embryonic stages using RT-PCR and found that the expression levels are higher than in wild-type mice. *Pitx3* over-expression in the lens during developmental stages was also confirmed at the protein level in the microphthalmos mutants via immunohistochemical analyses. Although lens fiber differentiation was not observed in the mutants, strong PITX3 protein signals were observed in the lens vesicles of the mutant lens. Thus, we speculated that abnormal PITX3, which lacks the C-terminus (including the OAR domain) as a result of the nonsense mutation, is expressed in mutant lenses. We showed that the expression of the downstream genes *Foxe3*, *Prox1*, and *Mip* was altered because of the *Pitx3* mutation, with large reductions in the lens vesicles in the mutants. Similar profiles were observed by immunohistochemical analysis of these proteins. The expression profiles of crystallins were also altered in the mutants. Therefore, we speculated that the microphthalmos/aphakia in this mutant is caused by the expression of truncated PITX3, resulting in the abnormal expression of downstream targets and lens fiber proteins.

## Introduction

Vertebrate eye formation is contingent on the complex interactions of transcription factors that regulate the expression of target genes. The precise temporal regulation of these genes is essential for normal eye development. Mutations in the genes encoding these transcription factors lead to severe congenital eye defects such as anophthalmia, aphakia and microphthalmia. These severe ocular diseases are found in approximately 30 of every 100,000 blind children worldwide. However, the precise pathogenesis and the optimal treatment protocols remain unclear [1], [2].

To date, several mutations in genes that mediate ocular development have been identified in humans [1], [2]. One of these genes, the paired-like homeodomain transcription factor 3 gene (*PITX3*), has a critical role in normal lens development. *PITX3* was first cloned as a homolog member of the PITX/RIEG homeobox family [3]. The PITX/RIEG family comprises the following three genes: *PITX1*, *PITX2* and *PITX3*. These genes encode a *bicoid*-related subclass of homeodomain proteins that play key roles in the development of various organisms [3], [4]. Moreover, all *Pitx*/*Rieg* genes have a highly conserved C-terminal region that has been termed the otp, aristaless, and rax (OAR) domain. In *PITX2*, this domain functions as an intrinsic inhibitor of DNA-binding activity mediated by protein-protein interactions [4].

In humans, *PITX3* mutations were first reported to play a role in dominant anterior segment mesenchymal dysgenesis (ASMD) and cataracts. Semina *et al*. [3] reported that a frameshift (p.Gly220ProfsX94) mutation in the C-terminus of the homeodomain caused by a 17-bp insertion/duplication (c.656_657ins17 or c.639_656dup17) leads to the onset of dominant ASMD [3], and a p.13Ser>Arg mutation in the homeodomain causes dominant cataracts [5]. Additionally, recessive microphthalmia caused by a 640_656del mutation (Ala214ArgfsX42) was recently reported in consanguineous populations [6]. Thus, *PITX3* mutations induce various ocular defects, and these defects are inherited via both dominant and recessive modes in human patients. Additionally, two *Pitx3* mouse mutant strains have been established. The *aphakia* (*Pitx3^ak^*) mouse is a recessive mutant that is characterized by the absence of a lens [7]. Double deletion of 652-bp and 1423-bp in the upstream region containing exon 1 and intron 1 of *Pitx3* has been identified as a causative mutation in *Pitx3^ak^*, and this mutation leads to the loss of *Pitx3* transcript expression [5], [8]. The eyeless (*Pitx3^eyl^*) homozygous mutant has closed eyelids, a thickened cornea and a rudimentary lens with some adhesion to the cornea. These phenotypes are caused by a guanine insertion (c.415_416insG) in *Pitx3* [9]. The c.415_416insG is predicted to cause a frameshift mutation resulting in the synthesis of 121 extra amino acid residues and generating a new stop codon at amino acid position 260. This mutation might synthesize a truncated protein that lacks the OAR domain, a known functional domain in the PITX/RIEG homeobox family [9].

Recently, we identified a novel spontaneous microphthalmia and aphakia (*miak*) mouse in a KOR1/Stm strain colony derived from the Japanese wild mouse *Mus musculus molossinus* [10]. In the present study, we report that a novel nonsense mutation located outside the homeodomain in *Pitx3* causes microphthalmia and aphakia in *miak* mice. The findings of this study also suggest that the *miak* mutant phenotypes caused by the expression of the truncated PITX3 protein differ from the phenotypes of the known null *Pitx3^ak^* mutation.

## Materials and Methods

### Ethics Statement

All of the procedures involving animals met the guidelines described in the Proper Conduct of Animal Experiments, as defined by the Science Council of Japan, and were approved by the Animal Care and Use Committee on the Ethics of the Tokyo University of Agriculture (Approval number: 250058) and the Tokyo Metropolitan Institute of Medical Science (Approval number: 13036 and 14081).

### Mice husbandry

The recessive *miak* mutation was first identified in a litter of the KOR1/Stm (KOR1) inbred strain at the Research Institute for Clinical Oncology in the Saitama Cancer Center. The mutants were crossed to C57BL/6J (B6J) for 12–14 generations followed by sibling matings and maintained at the animal facilities of both the Tokyo University of Agriculture and Tokyo Metropolitan Institute of Medical Science. We used wild-type and *miak* mice in a B6J background except for the histological analysis at 6 weeks of age.

### Histological analysis and immunohistochemistry

The eyeballs were removed from mice and were fixed, dehydrated, embedded in paraffin, and sectioned (5 µm) as previously described [11]. After removing the paraffin, the sections were stained with haematoxylin-eosin.

The eyeball sections were used for immunostaining. The procedure for immunohistochemistry of paraffin sections was previously described [11], [12] except for the use of Can Get Signal Solution B (TOYOBO, Osaka, Japan) to dilute the primary and secondary antibodies. As shown in Table S1, the primary antibodies for PITX3, N-cadherin (CDH2), forkhead box E3 (FOXE3), major intrinsic protein of eye lens fiber (MIP), paired box 6 (PAX6), Prospero-related homeobox 1 (PROX1), αA-crystallin, αB-crystallin, β-crystallin and γ-crystallin used in this study were obtained commercially and had been characterized in previous studies [12]–[19].

### Linkage and haplotype analyses

The DNA samples from (KOR1 -*miak*/*miak* × B6J) F_2_ and (B6J-*miak*/*miak* congenic × B6J) F_2_ mice were typed for multiple microsatellite markers located throughout the mouse genome. The markers were selected from the Microsatellite Database of Japan (http://www.shigen.nig.ac.jp/mouse/mmdbj/top.jsp) based on size variation between PCR products from B6J- and Japanese-derived strains (MSM/Ms and JF1Ms). The genotypes were then analyzed for cosegregation with the mutant phenotype, which is the easily identifiable characteristic of small eyes. The PCR conditions for genotyping were as previously described [20]. The polymorphisms of the PCR products were visualized on 4% agarose (3% Agarose XP and 1% Agarose S, Nippon gene, Tokyo, Japan) stained with ethidium bromide.

### Mutation analysis

The *miak* mutation in *Pitx3* was confirmed by DNA sequencing of the PCR products. A genomic fragment spanning the four coding exons of *Pitx3* was amplified from genomic DNA isolated from wild-type, *miak*/+ heterozygous and *miak*/*miak* homozygous mice of both KOR1 and B6J backgrounds. The Pitx3_F and Pitx3_R primers were used for amplification, and the following primers were used for sequencing: Pitx3_F1, Pitx3_F2, and Pitx3_F3 (Table S2). The PCR products were purified using the QIAquick Gel Extraction Kit (Qiagen, Valencia, CA), sequenced using a BigDye Terminator kit (Life Technologies, Grand Island, NY) and analyzed using an Applied Biosystems 3130×l Genetic Analyzer.

The PCR products were also amplified from DNA from the 20 (B6J-*miak*/*miak* × B6J) F_2_ mice, nine common inbred strains (B6J, 129X1/SvJ, A/J, BALB/cA, C3H/HeN, C57BL/6N, C57BL/10J, DBA/2J, NOD/Shi and SJL/J), and twelve wild-derived inbred strains (*M*. *m*. *domesticus*, PGN2/Ms, SK/Cam, WSB/Ei; *M*. *m*. *musculus*, CZEZHII/Ei, SWN/Ms; *M*. *m*. *molossinus*, KOR1, JF1/Ms, MOLF/Ei, MSM/Ms; and *M. m. castaneus*, CAST/Ei, HMI/Ms) using Pitx3_miak_F and Pitx3_miak_F primers (Table S2) to confirm the *miak* mutation. The PCR products were digested with *Sma*I (TOYOBO, Osaka, Japan), separated in a 2% agarose gel and stained with ethidium bromide.

### Whole mount *in situ* hybridization and quantitative RT-PCR

For *in situ* hybridization, digoxigenin (DIG) -labeled sense and antisense RNA probes were synthesized using a DIG RNA Labeling Kit (Roche Applied Science, Basel, Switzerland). The template was the coding sequence (nucleotide positions 34–720) that was amplified using the Pitx3_ISH_F and Pitx3_ISH_R primer pair (Table S2). The procedure for whole mount *in situ* hybridization was previously described [11].

For quantitative RT-PCR (qRT-PCR), the total RNA was isolated from embryonic (E) 11.5, E12.5 E14.5 and postnatal 30 (P30) eyes and P30 olfactory bulb using the RNeasy Mini Kit (Qiagen) following the manufacturer's protocol. The DNase-treated total RNA was reverse-transcribed using the Superscript VILO cDNA Synthesis Kit (Invitrogen). The *Pitx3*, *Prox1*, *Foxe3*, *Mip*, *Cryaa*, *Cryab*, *Cryba1* and *Cryga* transcripts were amplified and quantified using the 7500 Fast Real-Time PCR System (Life Technologies). The primers and kit used for the detection of these transcripts are shown in Table S2. These signal values were normalized to the *Gapdh* median signals, and the geometric means of target signals were calculated in triplicate.

### Electrophoretic mobility assay (EMSA)

For EMSAs, nuclear proteins were extracted from 12–14 whole eyeballs from B6J and B6J-*miak*/*miak* mice at E17.5 using a NE-PER Nuclear and Cytoplasmic Extraction Reagents Kit (Thermo Fisher Scientific, Rockford, IL) following the manufacturer's instructions. The oligonucleotides for the *Foxe3* enhancer and *Mip* promoters used Fox3-1-EMS (AAT CCC TGG CCA TTA ATC CCT CCT GCC AGC CC) and bcd1 (CTG CCC CTC CCA GGG ATT AAG AGT CCT CTA TA), respectively, as described by Ahmad et al. [16] and Sorokina et al. [21]. EMSAs were performed with a DIG Gel Shift Kit (Roche Applied Science). After a binding assay following the manufacturer's protocol, DNA-protein complexes were electrophoresed on 8% non-denaturing polyacrylamide gels in 0.25× TBE buffer and were blotted onto Hybond N+ (GE Healthcare Life Science, Piscataway, NJ). The DIG-label detection was performed according to the kit protocol.

### Statistical analysis

All results are presented as the mean ± standard deviation (SD). The differences among the multiple groups were analyzed by a one-way ANOVA with Tukey's post hoc multiple comparison test. The two groups were compared using Welch's t-test. GraphPad Prism 5 (GraphPad, San Diego, CA) was used to calculate column statistics and compute *P* values.

## Results

### The *miak* mouse mutant phenotype

**Figure 1:**
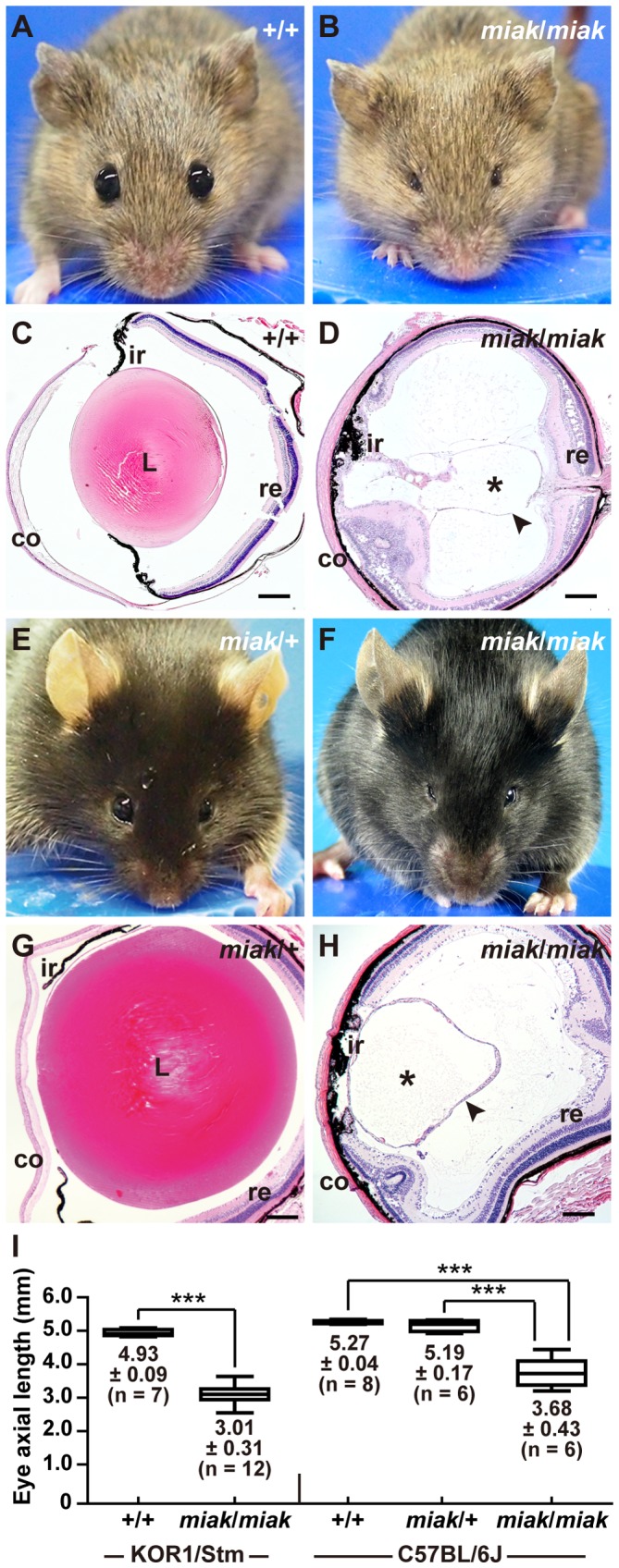
New spontaneous microphthalmia and aphakia (*miak*) mutations in mice. **A–D.** The visible phenotypes of the KOR1/Stm (**A**) and KOR1/Stm-*miak*/*miak* homozygous mouse (**B**). Comparison of the histological phenotypes between the wild-type (**C**) and *miak*/*miak* (**D**) mice at 6 weeks of age in the KOR1/Stm genetic background. The *miak* mutant mouse shows small eyes without severely closed eyelids (**B**). A lens section of the *miak* mouse shows the large fraction of crystallin lens (*) with a tissue-like lens capsule (arrowhead) not present in the eye (**D**). co, cornea; L, lens; ir, iris; re, retina. Scale bar * = *100 µm. **E–H.** Gross appearance (**E, F**) and histological features (**G, H**) of the *miak*/+ heterozygous (**E, G**) and *miak*/*miak* homozygous (**F, H**) mouse in the C57BL/6J background. In the *miak*/*miak* mice, the eye phenotypes are identical in the KOR1/Stm (**B, D**) and C57BL/6J (**F, H**) backgrounds. **I**. Quantitative analysis of the eye axial length in wild-type (+/+) and *miak*/*miak* mice in KOR1/Stm and +/+, *miak*/+ and *miak*/*miak* in C57BL/6J background at 6 weeks of age. ***P<0.001.

The KOR1-*miak* mutant had very small eyes (Figure 1B) compared to the eyes of the wild-type mice (Figure 1A); however, the eyelids of the mutants were obviously opened. To define the histological defects in the lenses of *miak* mutants, sagittal sections of the eye were prepared from wild-type (Figure 1C) and *miak* mutants (*miak*/*miak* homozygotes, Figure 1D). Via histological analysis, no lens fibers were detected in the *miak* mutants; however, the cornea, iris, lens capsule and retina were observed in the mutant eye sections (Figure 1D). Therefore, the small eye of KOR1-*miak* was histologically characterized as microphthalmia with the absence of a crystallin lens, namely aphakia. We established *miak*-congenic mice in the B6J genetic background, which is a common inbred strain. The B6J-*miak*/*miak* homozygotes showed microphthalmia (Figure 1F) and aphakia (Figure 1H), but the lenses of B6J-*miak*/+ heterozygotes were phenotypically normal (Figure 1E, G). The reduction ratio of the eye size in the B6J-*miak*/*miak* homozygotes (69.8–70.9%) compared to the wild-type (+/+ and *miak*/+ heterozygotes) mice is similar to the ratio calculated when the mice were in the KOR1 background (61.1%) (Figure 1G). Therefore, both microphthalmia and aphakia of the KOR1-*miak* mice were stably transmitted to the B6J-congenic mice.

**Figure 2:**
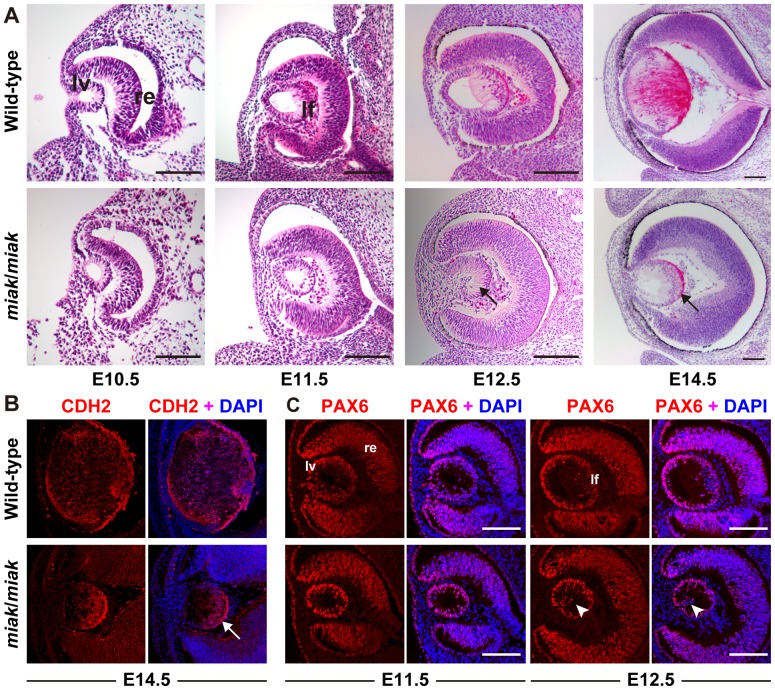
Histological analysis in the wild-type and *miak/miak* mice at embryonic stages. **A.** Hematoxylin-eosin staining of eye sections prepared from wild-type and *miak*/*miak* mice at E10.5, E11.5, E12.5 and E14.5. Although the lens vesicle (lv) and retina (re) are normally closed at E10.5 and E11.5, lens fiber (lf) differentiation was delayed in the *miak*/*miak* mice (arrows). **B.** The CDH2 labeling of the lens fiber at E14.5 confirming the delay of the lf differentiation in *miak* mice (arrow). **C.** PAX6 expression in the embryonic eyes of wild-type and *miak*/miak mice at E11.5 and E12.5. The abundant expression of PAX6 was observed in the lv and neural re in both the wild-type and *miak*/*miak* mice. However, ectopic expression of PAX6 was observed in the lv of *miak*/*miak* mice at E12.5 (arrowhead). Scale bar * = *100 µm.

To identify developmental malformations, we histologically investigated the lens phenotypes in embryonic wild-type (B6J) and B6J-*miak*/*miak* homozygous mice (Figure 2). In the wild-type mice, the formation of the lens vesicle and the differentiation of the lens fiber were observed at E10.5 and E11.5, respectively (Figure 2A). Although the lens vesicle developed normally, the differentiation of the lens fiber cells was not observed in the homozygous *miak* mutants at E10.5 and E11.5. In the E12.5 embryo, a further differentiated primary lens fiber was observed in the wild-type mice (Figure 2A). In contrast, the elongated lens fiber cells were scarcely recognized in the *miak* mutants. At E14.5, the differentiation of lens fibers was observed in wild-type mice. In contrast, the differentiation of lens fibers was extremely delayed in the *miak*/*miak* mice. To confirm the delay of lens fiber differentiation, we investigated the expression of CDH2, a known lens fiber marker in wild-type and *miak* mice at E14.5 (Figure 2B). CDH2 signals were abundant in the anterior region of wild-type lens fibers. Although we detected CDH2 in *miak* lenses, most signals were observed at the posterior region in the developing lens. Therefore, the *miak* mutation led to the delayed differentiation of lens fiber cells (Figure 2B). In addition, we analyzed PAX6 expression, a lens precursor marker, to confirm lens vesicle formation (Figure 2C). PAX6 expression was observed in the lens vesicle and retina in both wild-type and *miak* eyes at E11.5. However, ectopic expression was visible in *miak* lens vesicles at E12.5, and these expression patterns were consistent with the *Pitx3^ak^* mutation [16].

### Identification of the *miak* mutation

**Figure 3:**
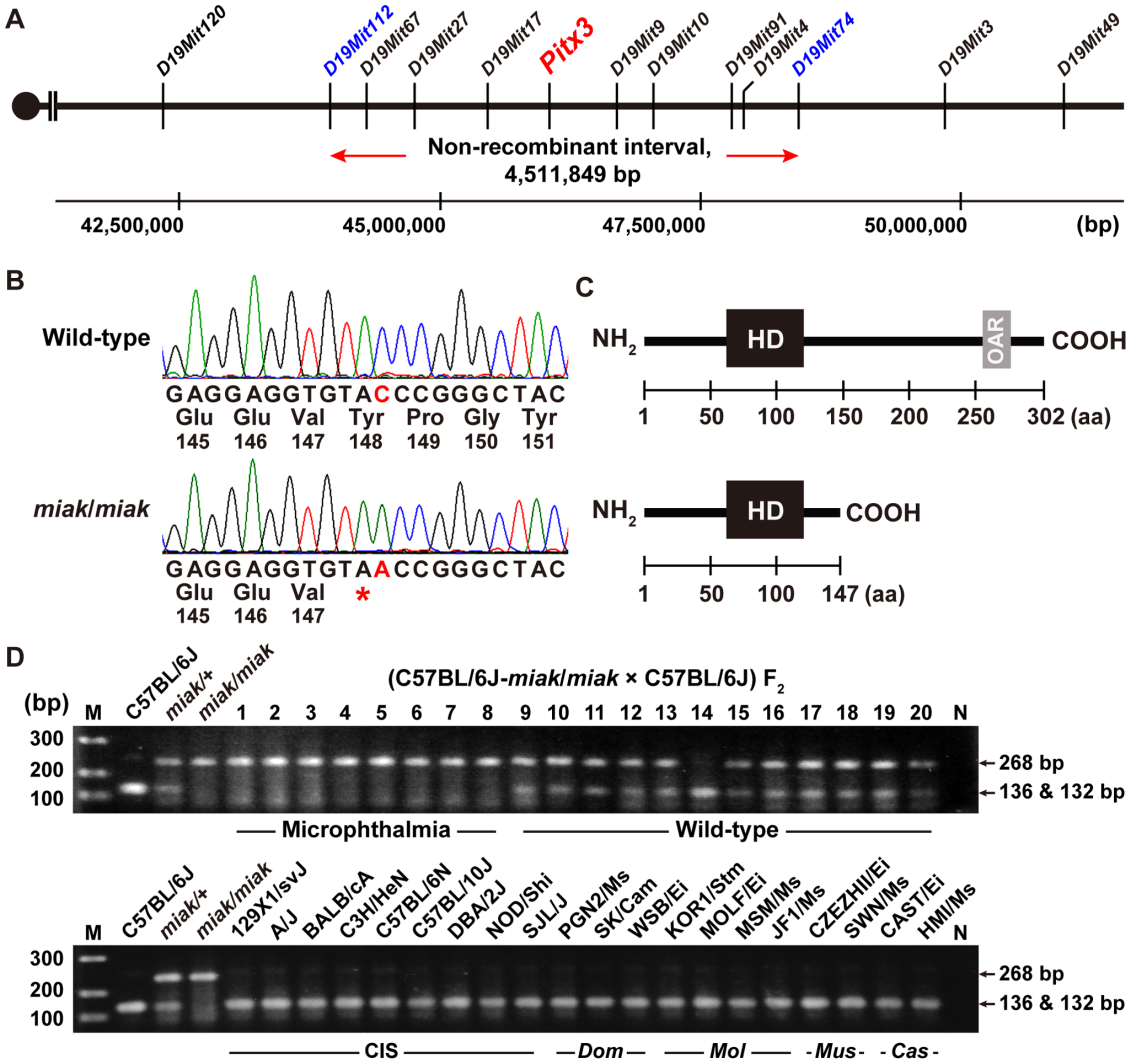
Positional cloning of the *miak* mutation. **A.** Genetic maps obtained by genotyping and phenotyping of the progeny from the intercross between (C57BL/6J-*miak*/*miak* congenic mice × C57BL/6J) F_2_. The blue markers *D19Mit 112* and *D19Mit74* define the non-recombinant interval containing *miak* mutation and *Pitx3* that is responsible gene for the mouse *Pitx3^ak^* and *Pitx3^eyl^* mutation [5], [9]. Distances on chromosome 19 are according to the mouse mm 10 (Genome Reference Consortium GRCm38) genomic sequence. **B.** Mutation analysis of *Pitx3* in the *miak* mouse. *miak* mice have a c.444C>A nonsense mutation in *Pitx3*. **C.** Schematic diagram of the domain structure of the PITX3 protein in the +/+ and *miak* mice. The domain structures were predicted by the SMART program, and the numbering of the amino acids (aa) is according to the PITX3 aa sequence of the wild-type and *miak* mice (NP_032878 and AB971349). PITX3 possesses homeodomain (HD, black box) and otp, aristaless, and rax (OAR, light gray box) as major functional domains near the N- and C-termini, respectively. The nonsense mutation in the *miak* mutants cause truncations of the PITX3 protein that result in a missing C-terminal OAR domain. **D.** The *miak* mutation disrupts a *Sma*I restriction site (CCCGGG) in *Pitx3*. The digestion of amplicons from wild-type mice produces bands at 136 and 132 bp. However, *miak/miak* mice are homozygous for the disruption of the *Sma*I site and yield only a single 268 bp band, whereas the *miak*/+ mice are heterozygous for the mutation as shown by the two banding patterns superimposed on one another. The top and bottom panels show the RFLP patterns of (C57BL/6J-*miak*/*miak* congenic mice × C57BL/6J) F_2_ progeny and wild-type inbred strains, respectively. M, marker (100 bp ladder); N, negative control (DDW); CIS, common inbred strain; *Dom*, *domesticus*; *Mol*, *molossinus*; *Mus*, *musculus*; *Cas*, *castaneus*.

To identify the responsible gene mutation of *miak*, we performed linkage analysis using 52 affected (KOR1-*miak*/*miak* × B6J) F_2_ mice and identified two markers, *D19Mit17* (46.772 Mb) and *D19Mit10* (47.152 Mb), on chromosome 19. Next, we genotyped eight affected and twelve unaffected (B6J-*miak*/*miak* × B6J) F_2_ mice. As expected, we detected the haplotype blocks from the B6J/B6J, B6J/KOR1 and KOR1/KOR1 genotypes in the F_2_ mice on chromosome 19 (Figure S1) and mapped the *miak* mutation to an approximately 4.5 Mb interval between the markers *D19Mit112* and *D19Mit74* (Figure 3A). Although 39 genes are located within this region, *Pitx3* was the strongest candidate gene because the *Pitx3^ak^* and *Pitx3^eyl^* mutations also localize to this candidate interval region [5], [9], and we therefore first screened for the *miak*-specific mutation in this gene. We sequenced the open reading frame of *Pitx3* and identified a c.444C>A mutation in *miak* mice (Figure 3B). The c.444C>A mutation is a nonsense mutation, which changes a tyrosine residue to a termination codon at amino acid 148 of the PITX3 protein (p.147Tyr>X). The PITX3 protein has two functional domains, the homeodomain and the OAR domain located in the in the N- and C-terminus, respectively (Figure 3C). The p.147Tyr>X mutation detected in the *miak* mice was located outside of the homeodomain, and we predicted that the mutation leads to a truncated PITX3 protein lacking the C-terminal after the 148^th^ residue or a functional null by nonsense mutation-mediated RNA decay [22]. We confirmed that the *miak* mutation cosegregates in mutant mice. The genotypes were easily identified by PCR-RFLP using the *Sma*I restriction enzyme. The genotypes from (B6J-*miak*/*miak* × B6J) F_2_ mice typed by PCR-RFLP analysis correlated with the mutant phenotypes (Figure 3D, top). Moreover, the RFLP in *miak* was not present in other mouse strains, including *Mus musculus domesticus-*, *M. m. musculus-*, *M. m. molossinus-*, and *M. m. castaneus-*derived inbred strains (Figure 3D, bottom).

### Expression of *Pitx3* gene and protein in wild-type and *miak* mutant mice

**Figure 4:**
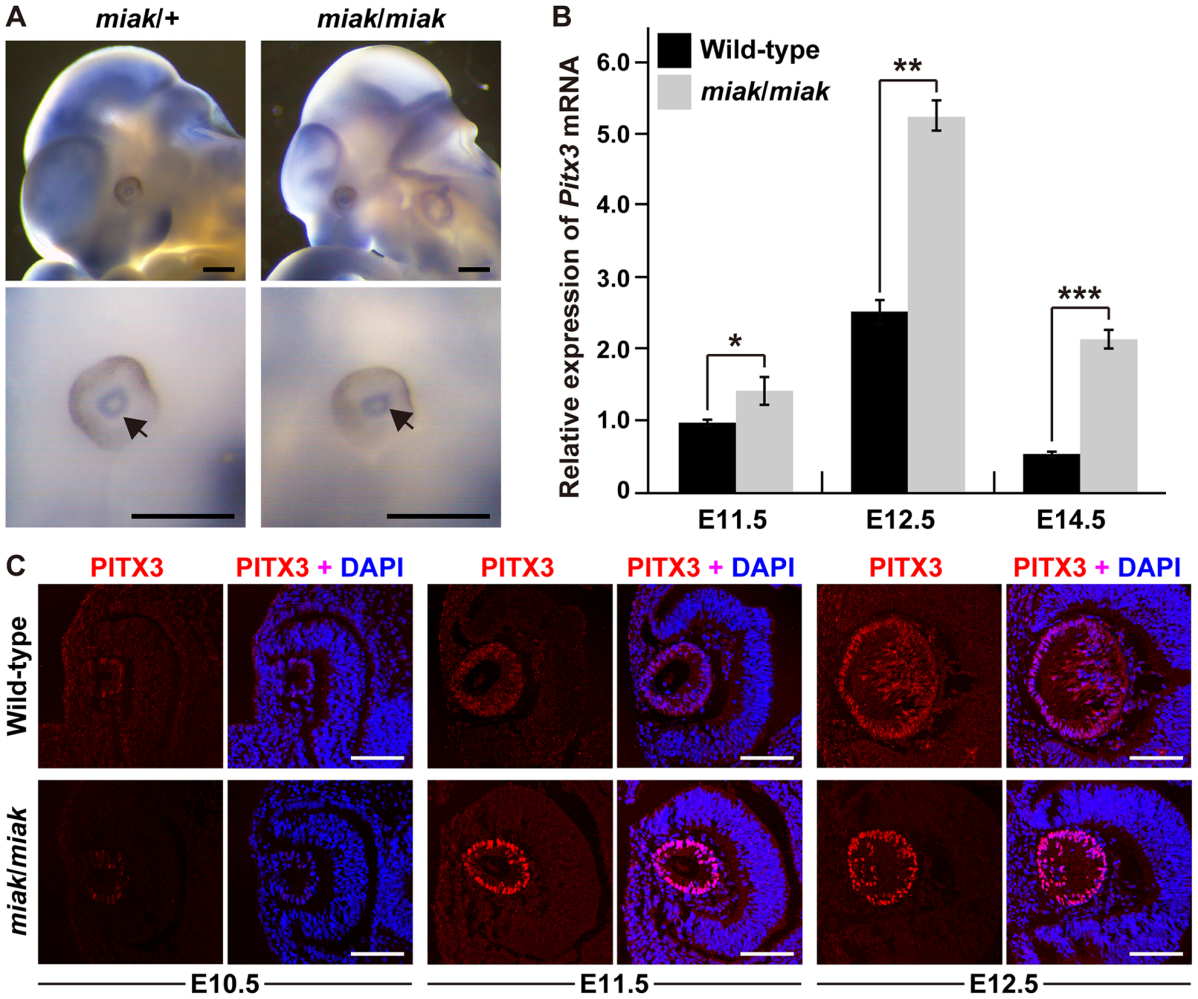
Expression analyses of *Pitx3* transcripts and PITX3 protein in wild-type and *miak* mice. **A.** Whole-mount *in situ* hybridization analysis in *miak*/+ and *miak*/*miak* in E11.5 embryos. The bottom panels show a magnified image of the eyes compared to the top panels. The arrows indicate the expression of *Pitx3*, detected as blue signals. Scale bar  = 500 µm. **B.** The relative levels of *Pitx3* mRNA in the eyes of wild-type (+/+) and *miak*/*miak* mice at E11.5, E12.5 and E14.5. *Pitx3* mRNA expression was measured by real-time RT-PCR analysis using the Mm_Pitx3_1 primer set (Table S1). The values shown in the graph indicate the mean relative expression levels and the SDs of triplicate eye mRNAs. The expression levels in wild-type mice at E11.5 were assigned an arbitrary value of 1 for comparative purposes. ns, no significant differences; *P<0.05; ***P<0.001. **C.** Over-expression of PITX3 protein during lens development in *miak* mice. Confocal images show the lenses double-labeled with PITX3 antibody (red) and DAPI (blue) in wild-type and *miak*/*miak* mice at E10.5, E11.5 and E12.5. Strong PITX3 signals were observed in the *miak*/*miak* mice at E11.5 and E12.5 compared to the wild-type mice. Scale bar * = *100 µm.

**Figure 5:**
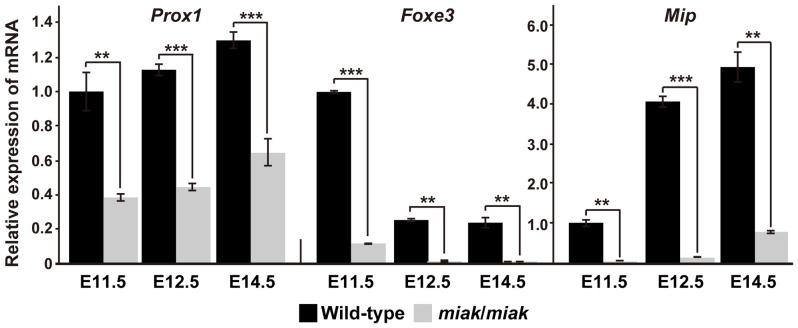
mRNA reduction of the downstream targets of PITX3 in *miak* mice at embryonic stages. The relative levels of *Prox1*, *Foxe3*, and *Mip* mRNA in the eye of wild-type (+/+) and *miak*/*miak* mice at E11.5, E12.5 and E14.5. The mRNA expression levels were measured by real-time RT-PCR analysis using specific primer sets (Table S1) for each gene. The values shown in each graph indicate the mean relative expression levels and the SDs of triplicate eye mRNAs. The expression levels in wild-type mice at E11.5 were assigned an arbitrary value of 1 for comparative purposes. **P<0.01; ***P<0.001.

To investigate the effects of the *miak* mutation on the expression of *Pitx3* transcripts, we performed whole-mount *in situ* hybridization analysis in E11.5 embryos to define *Pitx3* expression and localization. *Pitx3* was robustly expressed in the eyes and brain, as shown in a previous report (Figure 4A) [23]. The expression level and localization of the *Pitx3* transcript in the *miak* embryo was identical to the *miak*/+ heterozygote. However, we detected expression changes in *Pitx3* at the RNA level between the wild-type and *miak* mutant mice using qRT-PCR analysis. The analysis revealed that eye RNAs from the *miak* mutant mice show significantly higher expression than wild-type mice, and *Pitx* was up-regulated by ∼2.5-fold at E11.5 and E12.5 in the mutant mice (Figure 4B). To confirm the predicted corresponding increase in PITX3 protein levels in the eye of *miak* mutants, we performed expression analyses using a goat polyclonal anti-PITX3 antibody, whose specificity was confirmed via the loss of signal in the *Pitx3^ak^* mutant in a previous study [16]. We unfortunately failed to detect a specific-band for PITX3 in the mouse eye protein extracts by immunoblot using this antibody. In contrast, we observed immunofluorescence for PITX3 in the lens vesicles as reported in a previous immunohistochemical study [16]; however, the signal was faint in the lenses from the wild-type and *miak* mutant mice at E10.5, respectively (Figure 5). At E11.5 and E12.5, the PITX3 signals gradually increased in the lenses in all of the genotypes. Although the lens localization of PITX3 was similar in the wild-type and *miak* mutant mice, the immunofluorescence was more abundant in the *miak* mice (Figure 5). Therefore, we hypothesized that the mutated PITX3, which lacks the C-terminus including the OAR domain due to the p.147Tyr>X mutation, in the lens of the *miak* mutants leads to the overexpression of PITX3.

### Expression of the downstream targets of PITX3 in wild-type and *miak* mutant mice

To date, several downstream genes regulated by PITX3 have been reported, and their expression was down-regulated in lenses of *Pitx3^ak^* mutants during development [16], [23], [24]. To verify that the *miak* phenotype is caused by the *miak* mutation and the expression of truncated PITX3, we investigated the expression profiles of PITX3 downstream molecules in the eyes of the wild-type and *miak* mice at E11.5, E12.5, and E14.5 using qRT-PCR analysis. The expression levels of two transcription factors, *Prox1* and *Foxe3*, which have roles in normal lens development, are altered by the *Pitx3^ak^* mutation [16], [23]. Both *Prox1* and *Foxe3* were significantly reduced in *miak* mice compared to wild-type mice (Figure 5). Next, we investigated the expression of *Mip*, also known as aquaporin 0 (AQP0) and the most abundant membrane protein in the lens fiber, because its expression is directly regulated by the PITX3 homeodomain [21]. In wild-type mice, the *Mip* transcript expression gradually increased during development (Figure 5). In contrast, the expression levels were markedly reduced in the *miak* mutant at all of the developmental stages (Figure 5).

**Figure 6:**
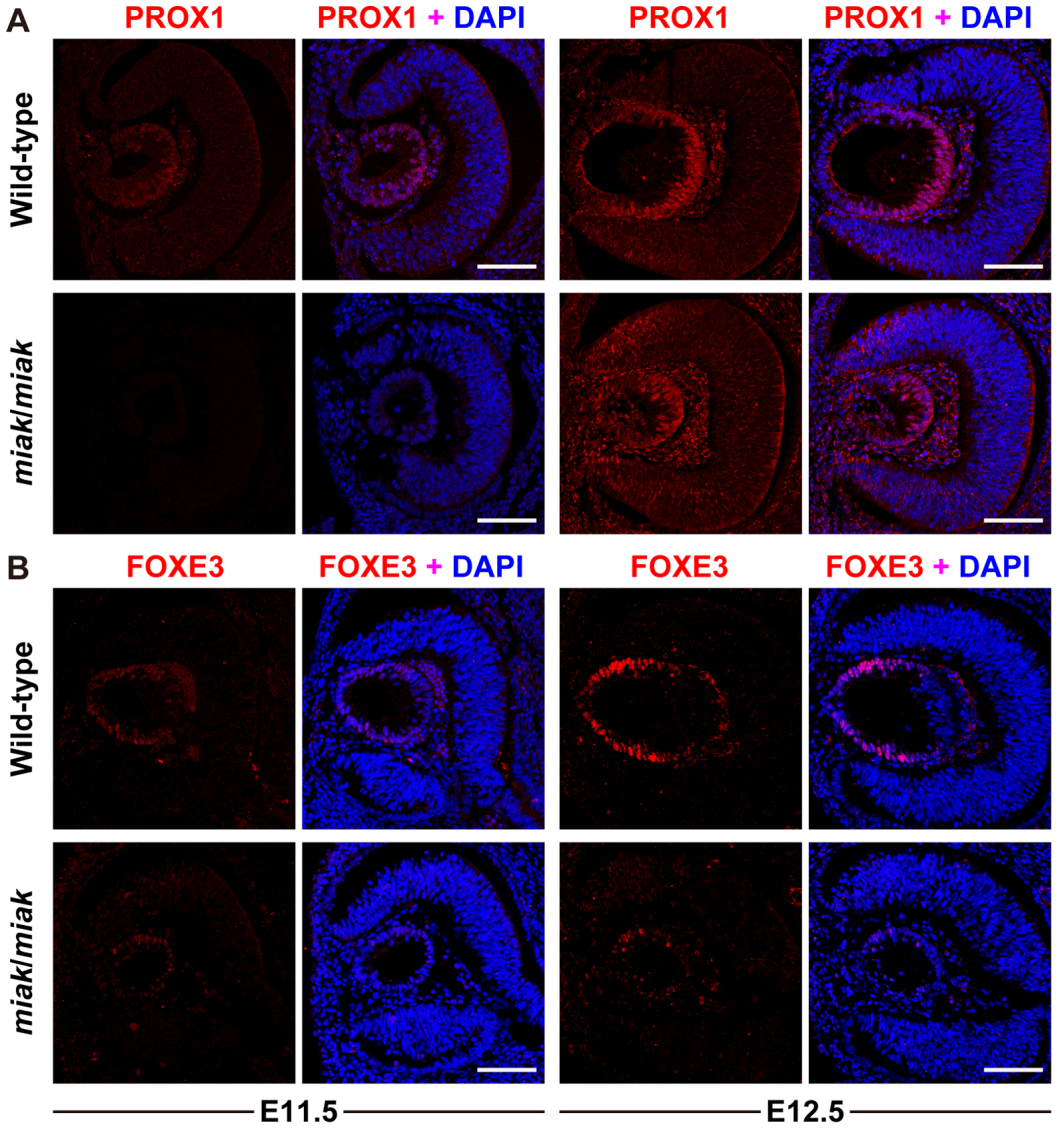
Localization and expression of the PROX1 (A) and FOXE3 (B) proteins during lens development in wild-type and *miak* mice at embryonic stages. Confocal images show the lenses double-labeled for protein (red) and DAPI (blue) in the wild-type (+/+) and *miak*/*miak* mice. Scale bar * = *100 µm. **A.** PROX1 labeling of the lens at E11.5 and E12.5. The delay and slight reduction of PROX1 signals was observed in *miak*/*miak* mice at both stages. **B.** FOXE3 labeling of the lens at E11.5 and E12.5. Immunohistochemistry reveals the dramatically reduced signals of FOXE3 in *miak*/*miak* mice.

**Figure 7:**
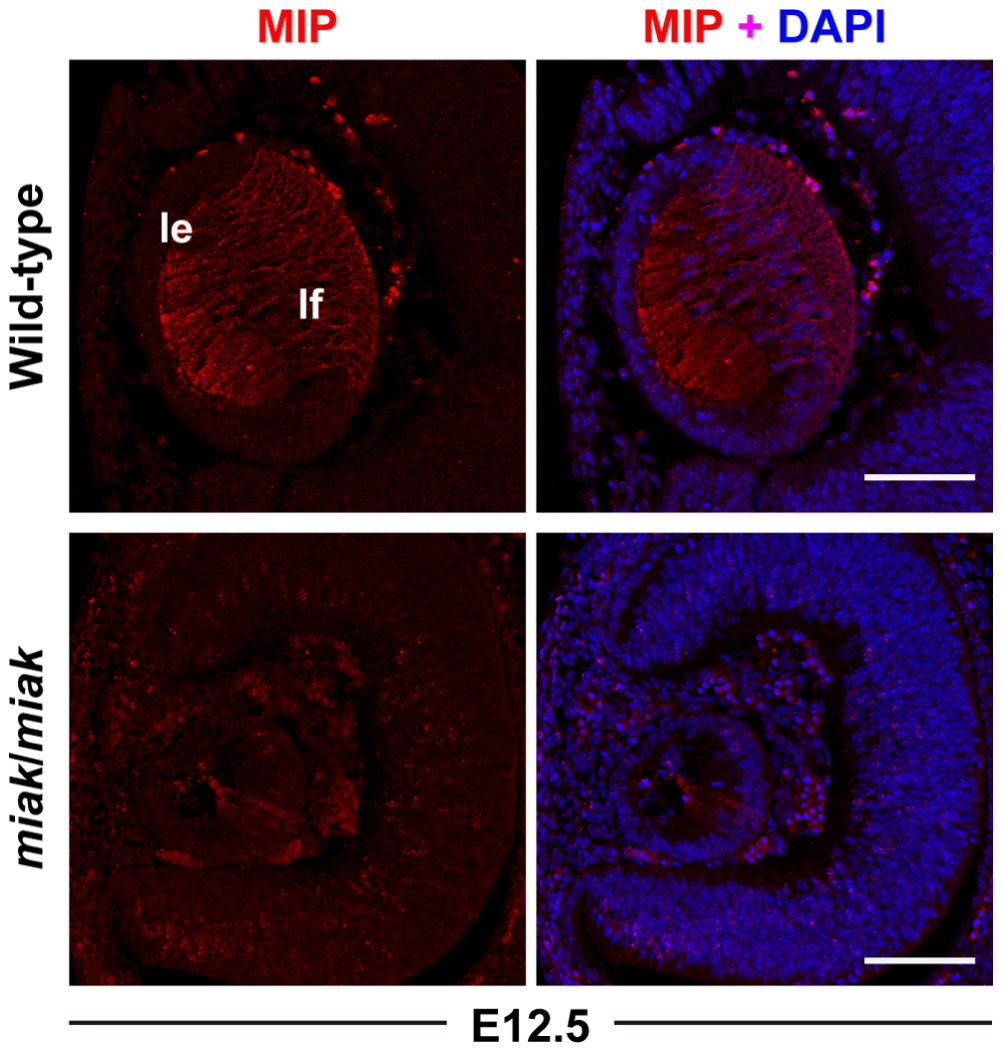
Localization and expression of the MIP proteins during lens development in wild-type and *miak* mice during embryonic stages. MIP labeling of the lens at E12.5. The MIP signals in lens fiber (lf) were regulated in *miak*/*miak* mice.

The reduction in the expression of the downstream targets of PITX3 was confirmed by immunohistochemistry. PROX1 was weakly expressed at E11.5 and was strongly expressed in the posterior region of lens vesicle in the wild-type mice at E12.5, which is consistent with previous studies [16], [23], [24] (Figure 6A). Although the expression patterns were similar in the wild-type and *miak* mutants, the signals were obviously reduced in the mutants (Figure 6A). Consistent with previous reports, FOXE3 expression was observed in the lens epithelium at E11.5 and E12.5 in the wild-type mice [16], [19], [24] (Figure 6B). Similar to PROX1, the *miak* mutants exhibited a weak FOXE3 expression pattern at E11.5 and E12.5 (Figure 6B). A reduction in the expression of MIP was also detected in the lens of the *miak* mutants, and low expression of MIP was observed in the posterior region of the lens vesicle in the *miak* mutants. By contrast, MIP was robustly expressed in the plasma membrane of the lens fiber cells in the wild-type mice (Figure 7). Thus, downstream targets of PITX3 were affected in *miak* mice, and these results suggest that these molecules are down-regulated by the expression of truncated PITX3 caused by the *miak* mutation.

**Figure 8:**
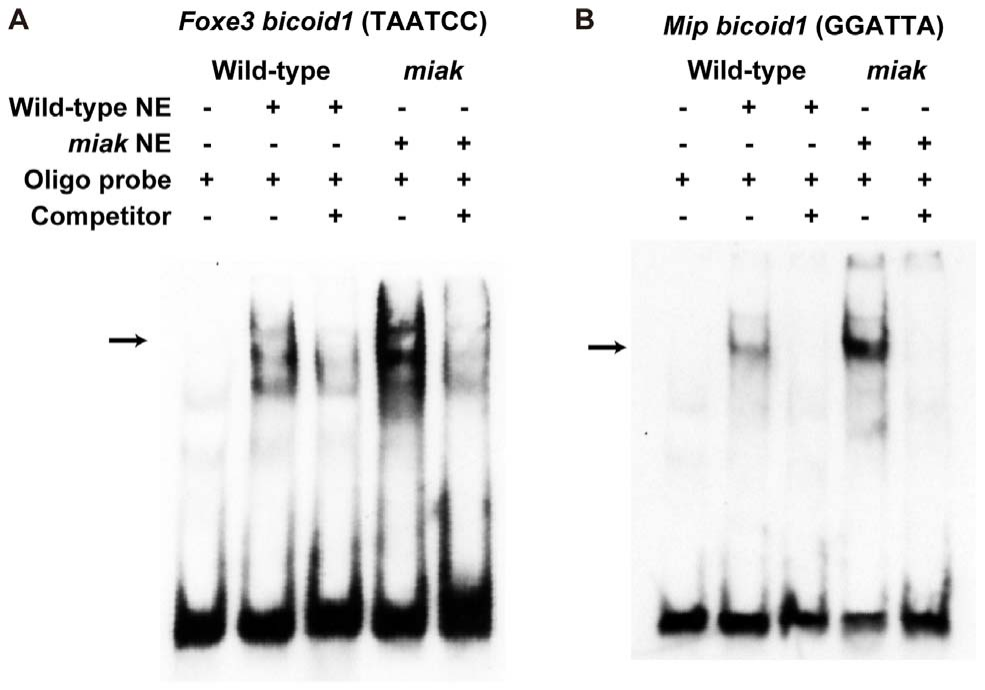
Evaluation of the interaction between *Foxe3-* (A) and *Mip-* (B) *bicoid* sites and proteins in wild-type and –*miak* mice by electrophoretic mobility assay (EMSA). EMSA performed with *Foxe3*- [16] and *Mip-* [21] *bicoid* oligonucleotides (oligo probe) and nuclear extracts (NE) from wild-type and *miak* eyes at E17.5. Although the formation of the specific EMSA complex occurred by combining oligo probes and NEs from wild-type and *miak* mice, the binding ability was increased with *miak*-NE and both *Foxe3*- and *Mip-bicoid* oligo probes. The binding ability of both oligo probes was inhibited by 10-fold excess unlabeled competitive probes (competitor).

To confirm whether the down-regulation of downstream targets was caused by the overexpression of truncated PITX protein, we investigated by EMSA the binding of nuclear extracts (NEs) from wild-type and *miak* mutant eyes with the well-characterized *Foxe3* enhancer [16] and *Mip* promoter [21] containing *bicoid* elements, which can interact with PITX3 protein. We observed the formation of specific EMSA complexes from the combination of *Foxe3* and *Mip* oligo probes and NEs from wild-type mice (Figure 8A, B). We also detected the binding of NEs from *miak* mice to both *Foxe3* and *Mip* oligo probes, and the binding reactivity was enhanced compared to the wild-type NEs (Figure 8A, B). This result suggested that the excess binding of truncated PITX3 protein to the *Foxe3* enhancer and *Mip* promoter may lead to down-regulation of FOXE3 and MIP in *miak* mutants.

### Expression of the crystallins in wild-type and *miak* mutant mice

**Figure 9:**
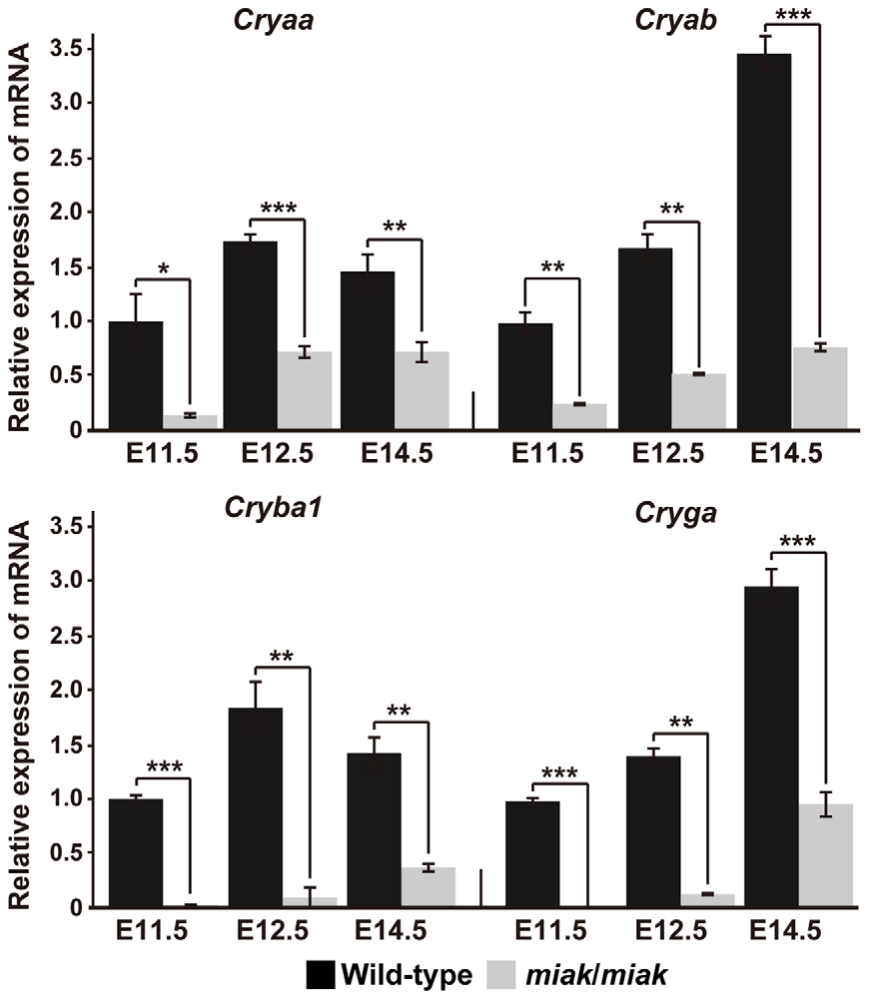
mRNA reduction of the crystallins in *miak* mice at embryonic stages. The relative levels of *Cryaa*, *Cryab*, *Cryb1* and *Cryga* mRNA in the eye of wild-type (+/+) and *miak*/*miak* mice at E11.5, E12.5 and E14.5. The mRNA expression levels were measured by real-time RT-PCR analysis using specific primer sets (Table S1) for each gene. The values shown in each graph indicate the mean relative expression levels and the SDs of triplicate eye mRNAs. The expression levels in wild-type mice at E11.5 were assigned an arbitrary value of 1 for comparative purposes. *P<0.05; **P<0.01; ***P<0.001.

We also performed qRT-PCR analysis of the three crystallin transcripts *Cryaa*, *Cryab*, *Cryb1*, and *Cryga.* The expression patterns of these molecules are useful for understanding the developmental state of the lens cells because the crystallins are the most abundant and stable lens proteins [23], [25], [26]. Significant reductions of the four crystallins were detected in the *miak* mice at all of the developmental stages (Figure 9).

**Figure 10:**
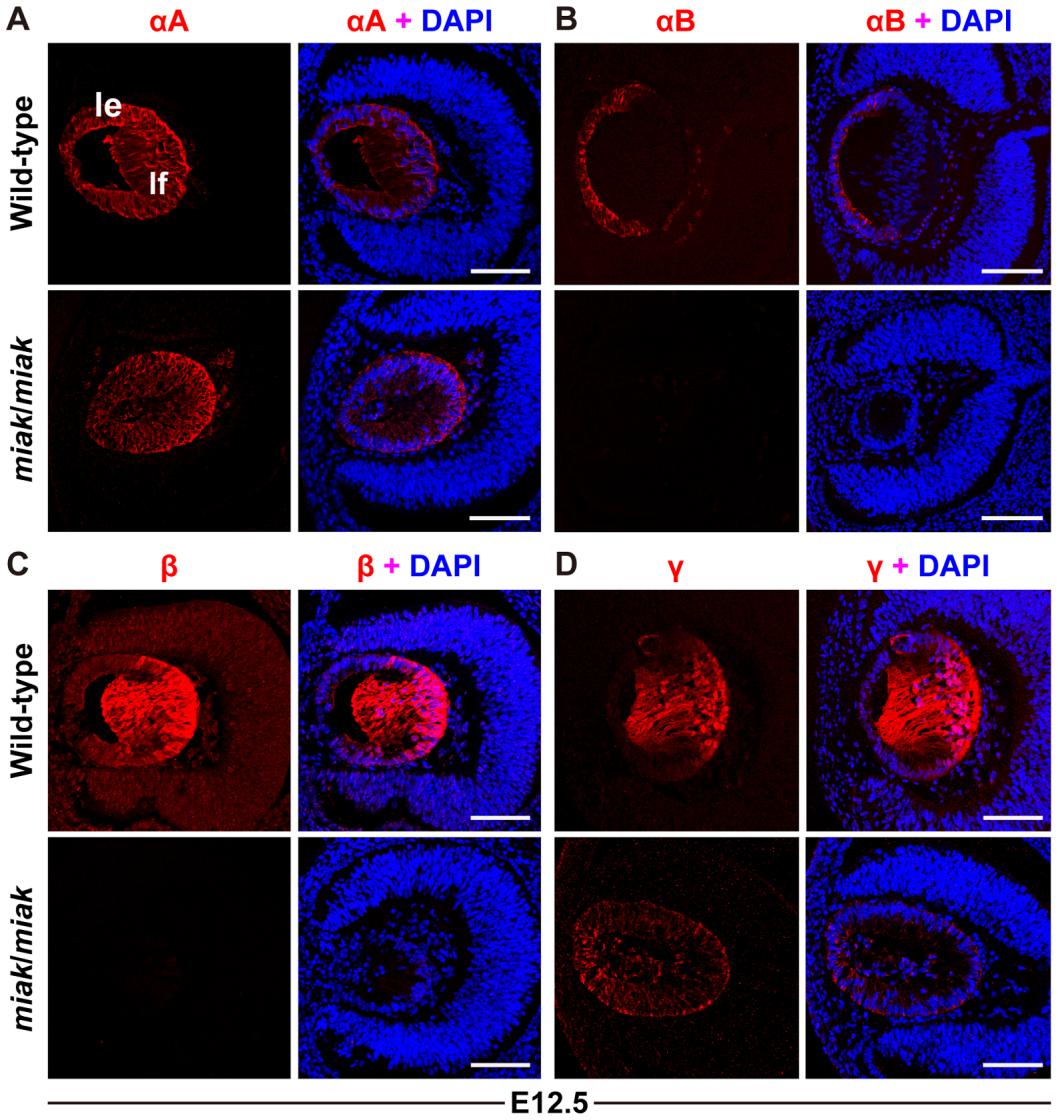
Down-regulation of the αA, αB, β and γ-crystallins caused by the *miak* mutation of *Pitx3*. Confocal images show the double-labeled crystallin proteins (red) and DAPI (blue) in the lenses of wild-type and *miak*/*miak* mice at E12.5. Scale bar * = *100 µm. **A.** The αA-crystallins labeling of the lens. The expression patterns of αA-crystallins were similar in the lens epithelium (le) and lens fiber (lf) of the wild-type and *miak* mice; however, the αA-crystallins signals may be slightly reduced in the *miak* mice. **B.** The αB-crystallins labeling of the lens. The αB-crystallin signals were not detected in the *miak*/*miak*. **C.** The β-crystallin labeling of the lens. The β-crystallin signals were barely detected in the *miak*/*miak* lens. **D.** The γ-crystallin labeling of the lens. Immunohistochemistry reveals the dramatically reduced γ-crystallin signals in the *miak* mice as well as ectopic expression in the anterior region of the lens.

Next, immunohistochemical analysis revealed that αA-crystallin was expressed in the lens vesicles of both the wild-type and *miak* mice, but the αA-crystallin signals were slightly reduced in the *miak* mutants (Figure 10A). In contrast, the αB-crystallin signals detected in the wild-type lens epithelium were scarcely observed in *miak* mutants (Figure 10B). Although β- and γ-crystallins were abundantly expressed in the lens fiber of the wild-type mouse, their expression was barely detected in the *miak* mouse at E12.5 (Figure 10C, D). The expression patterns of αB-, β- and γ-crystallins were similar to the *Pitx3^ak^* mutant; however, the αA-crystallin expression observed in the *miak* mutant differed from the known mutant [23]. Therefore, the lens vesicles may have developed normally, but the lens fiber cell differentiation may have been delayed in the posterior region of the lens vesicle in the *miak* mutants.

Ninkovic et al. [27] reported that αA -crystallin has a role in the survival of the dopaminergic olfactory bulb. To verify the effects of the *miak* mutation in this tissue, we attempted to detect crystallins in olfactory bulb in P30 adult mice. We first performed RT-PCR analysis of *Cryaa*, *Cryab*, *Cryb1* and *Crybb2* in the eye and olfactory bulb (Figure 2A). Although these crystallins were strongly expressed in the eye, the expression levels of all transcripts except *Cryab* were extremely low in the olfactory bulbs of both wild-type and *miak* mice (Figure S2A). We quantified *Cryab* transcript levels by qRT-PCR analysis in the wild-type and *miak* mutant olfactory bulbs (Figure S2B). The expression level of *Cryab* in *miak* mice was similar to wild-type. Immunohistochemistry of αB-crystallins revealed that both proteins were expressed in several neurons in the olfactory bulb (Figure S3C). Their expression patterns in *miak* mutants were identical to wild-type. Therefore, these findings suggest that *miak* mutation only affects the expression of crystallins in the early stage of lens development.

## Discussion

To identify crucial genes and modifier genes that regulate ocular development and disorders, the establishment of mouse models may be highly effective. Specifically, profound ocular diseases are genetically heterogeneous, with potentially overlapping phenotypes resulting from mutations in multiple genes and varying phenotypes caused by different mutations in a single gene [2]. Additionally, anophthalmia and microphthalmia show some evidence of familial recurrence but usually no clear Mendelian transmission pattern [28], which may be a reflection of several potentially interactive factors such as oligogenic causation, gene-environment interactions, and stochastic variations in development [29]. Therefore, mouse models that have allelic variants and varied genetic backgrounds in *Pitx3* will provide essential information for understanding the mechanisms of ocular pathogenesis and development.

In this study, we established and characterized a novel spontaneous *miak* mutant mouse with microphthalmia and a delay in lens fiber differentiation (Figure 1, 2). We identified a nonsense mutation, c.444C>A of *Pitx3*, which leads to p.148Tyr>X, and predicted that this mutation is a strong candidate to explain the microphthalmia and aphakia in the *miak* mutant (Figure 3). Furthermore, the over-expression of the PITX3 mutant protein truncated 155-aa of the C-terminus resulting from a nonsense mutation was confirmed in *miak* mutant mice (Figure 4C). We also investigated the lens phenotypes of *miak* heterozygous mice for potential gain-of-function and dominant negative effects of overexpression of truncated PITX3 by *miak* mutation. A point mutation (p.13Ser>Arg) and a truncation by frameshift mutation (p.Gly220ProfsX94) in *PITX3* cause dominant cataracts and ASMD, respectively, in humans [4]. In *miak*/+ heterozygous mice, the histological phenotypes of the lens and eye size were similar to wild-type mice (Figure 1E, G, Figure S3A). In addition, the transparency of lens was maintained until late stages in *miak/+* mice (Figure S3B). Thus, the *miak* mutant allele of *Pitx3* seems to hardly exert any dominant effects in lens phenotypes.

In *miak* mice, loss of the OAR domain of the PITX3 protein was predicted by a nonsense mutation (Figure 3C). The function of the PITX3 OAR domain has been investigated by *in vitro* assays using p.13Ser>Asn and p.Gly220ProfsX94 mutant proteins [30]. This previous study revealed that the PITX3 p.Gly220ProfsX94 mutation results in a partial loss of function and does not have a dominant negative effect. The p.Gly220ProfsX94 mutation located in the distal region of the homeodomain has been found in multiple pedigrees and causes dominant cataracts often accompanied by severe ASMD [30]. This variability of phenotype associated with the p.Gly220ProfsX94 mutation may be caused by the loss of the OAR domain. Although the functions of this domain are not yet fully understood, other OAR-containing proteins show that the OAR domain appears to perform an inhibitory function because the deletion of the domain results in increased DNA binding or the transactivation of target promoters [31]. Additionally, the C-terminal region of other PITX proteins has multiple regulatory roles and is involved in specific protein-protein interaction [32], [33]. In this study, we showed that the loss of the PITX3 OAR domain leads to the enhancement of PITX3 transcription and translation (Figure 4), which is most likely due to the obliteration of the anti-transcriptional activity associated with specific protein-protein interactions caused by the loss of the OAR domain.

In addition, we showed that the expression of the downstream targets of PITX3 protein was affected in the *miak* mutants (Figures 5–7). We detected an enhanced binding reactivity of nuclear extracts from *miak* mice, compared to wild-type nuclear extracts, to both *Foxe3* and *Mip* oligo probes, including *bicoid* elements, when assaying PITX3 binding EMSAs (Figure 8). Although we could not confirm whether the truncated PITX3 resulting from the *miak* mutation was included in the DNA-protein complex, this result suggests that the truncated PITX3 proteins can bind the *bicoid* elements of *Foxe3* and *Mip*. These results suggest that the OAR domain has a role in the positive regulation of the expression of the downstream genes. MIP/AQP0 protein, which acts on the maintenance of lens fiber cells mediated by water channel activities, is a direct transcriptional target of the PITX3 homeodomain in the lens [34]. A p.Gly220ProfsX94 mutation in PITX3 leads to a large reduction in *Mip* transcript [21]. This result revealed that the OAR domain as well as the homeodomain is required for the normal transcription of *Mip* and that *Mip* expression is inhibited by the loss of OAR domain. Therefore, the interaction between the OAR domain and homeodomain of PITX3 protein may be essential for the normal expression of downstream genes such as *Mip*, *Prox1* and *Foxe3*.

Moreover, Medina-Martinez et al. [23] reported the downregulation of the *Cry* genes in the lens of the *Pitx3^ak^* mutant. However, the expression of αA-crystallin was detected in the *miak* mutant (Figure 8). This difference in the expression pattern in αA-crystallin may represent variations in lens vesicle formation between *Pitx3^ak^* and *miak* mutants. α-crystallin is composed of two molecules, αA- and αB-crystallin, which are encoded by the *Cryaa* and *Cryab* genes, respectively. During the embryonic period, *Cryaa* begins to be expressed on the lens cup at E10-10.5, while *Cryab* is first detected at E9.5 in the mouse lens. The later expression of these genes primarily occurs on the lens fiber and lens epithelial cells [25], [35]. Therefore, we suggest that the early stages of *miak* eye development may be normal compared to the *Pitx3^ak^* mutant, and the presence or absence of the homeodomain in the PITX3 protein may cause the phenotypic variation observed between the *Pitx3^ak^* and *miak* mutants.

## Supporting Information

Figure S1 **Haplotype analysis of (C57BL/6J-** ***miak*** **/** ***miak*** **congenic mice × C57BL/6J) F_1_ mice on chromosome 19.** Polymorphic MIT markers for genotyping are shown on the left. The marker positions on chromosome 19 are according to the mouse mm 10 (Genome Reference Consortium GRCm38) genomic sequence. The number of offspring inheriting each type of chromosome is listed at the bottom of each column. The arrow indicates the non-recombinant interval containing the *miak* mutation.
(TIF)

Figure S2 **Expression analysis of αA and αB-crystallins in olfactory bulb in wild-type and** ***miak*** **mice.** **A.** Comparison of crystallin (*Cryaa*, *Cryab*, *Cryb1* and *Crybb2*) expression between wild-type and *miak*/*miak* mice at P30 by RT-PCR. cDNA integrity was confirmed with *Gapdh* control band (bottom panel). **B.** Relative expression level of *Cryab* transcript in olfactory bulb of wild-type and *miak*/*miak* at P30. The values shown in each graph indicate the mean relative expression levels and the SDs of triplicates. The expression levels in wild-type olfactory bulbs were assigned an arbitrary value of 1 for comparative purposes. n.s. no significant difference. **C, D.** Immunohistochemistry of αA-crystallin (**C**) and αB-crystallin (**D**) in olfactory bulb of wild-type (top) and *miak*/*miak* (bottom) mice at P30. The right panels indicated higher magnified images of periglomerular layer (pgl). gl, glomerular layer; epl, external plexiform layer. Scale bar * = *100 µm.
(TIF)

Figure S3 **Lens phenotypes in** ***miak*** **/+ heterozygous mice.** **A.** Highly magnified image of the cornea and lens epithelium from the lens section in *miak*/+ mouse at 6 weeks of age. co, cornea; ale, anterior lens epithelium; lfc, lens fiber cell. Scale bar * = *100 µm. **B.** Dark field imaging of the dissected lens from *miak*/+ mouse at 10 months of age. The procedure for phenotyping was previously described [11]. The *miak*/+ mice show normal transparency. Scale bar * = *500 µm.
(TIF)

Table S1 **Primary antibodies used in this study.**
(XLSX)

Table S2 **Information regarding the sequencing, PCR-RFLP, RT-PCR (qRT-PCR) and cRNA probe synthesis for in situ hybridization.**
(XLSX)
