# Supplementary figures and images for: Expression of Truncated PITX3 in the Developing Lens Leads to Microphthalmia and Aphakia in Mice
Source: PLoS One. 2014 Oct 27;9(10):e111432. doi: 10.1371/journal.pone.0111432 (PMC4210183; doi:10.1371/journal.pone.0111432)

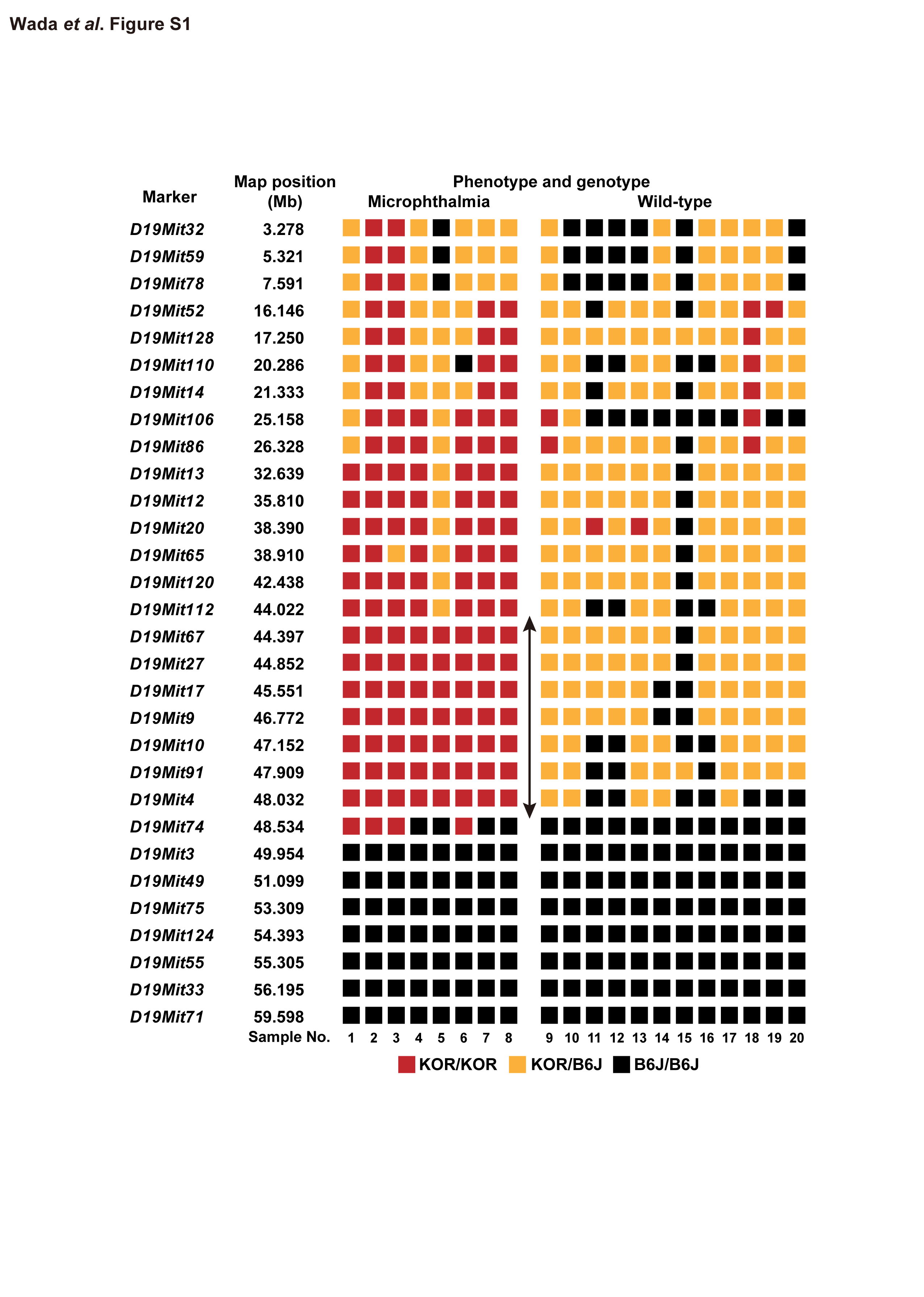

Supplement: Figure S1 — Haplotype analysis of (C57BL/6J- miak / miak congenic mice × C57BL/6J) F1 mice on chromosome 19. Polymorphic MIT markers for genotyping are shown on the left. The marker positions on chromosome 19 are according to the mouse mm 10 (Genome Reference Consortium GRCm38) genomic sequence. The number of offspring inheriting each type of chromosome is listed at the bottom of each column. The arrow indicates the non-recombinant interval containing the miak mutation. (TIF) [file pone.0111432.s001.tif]

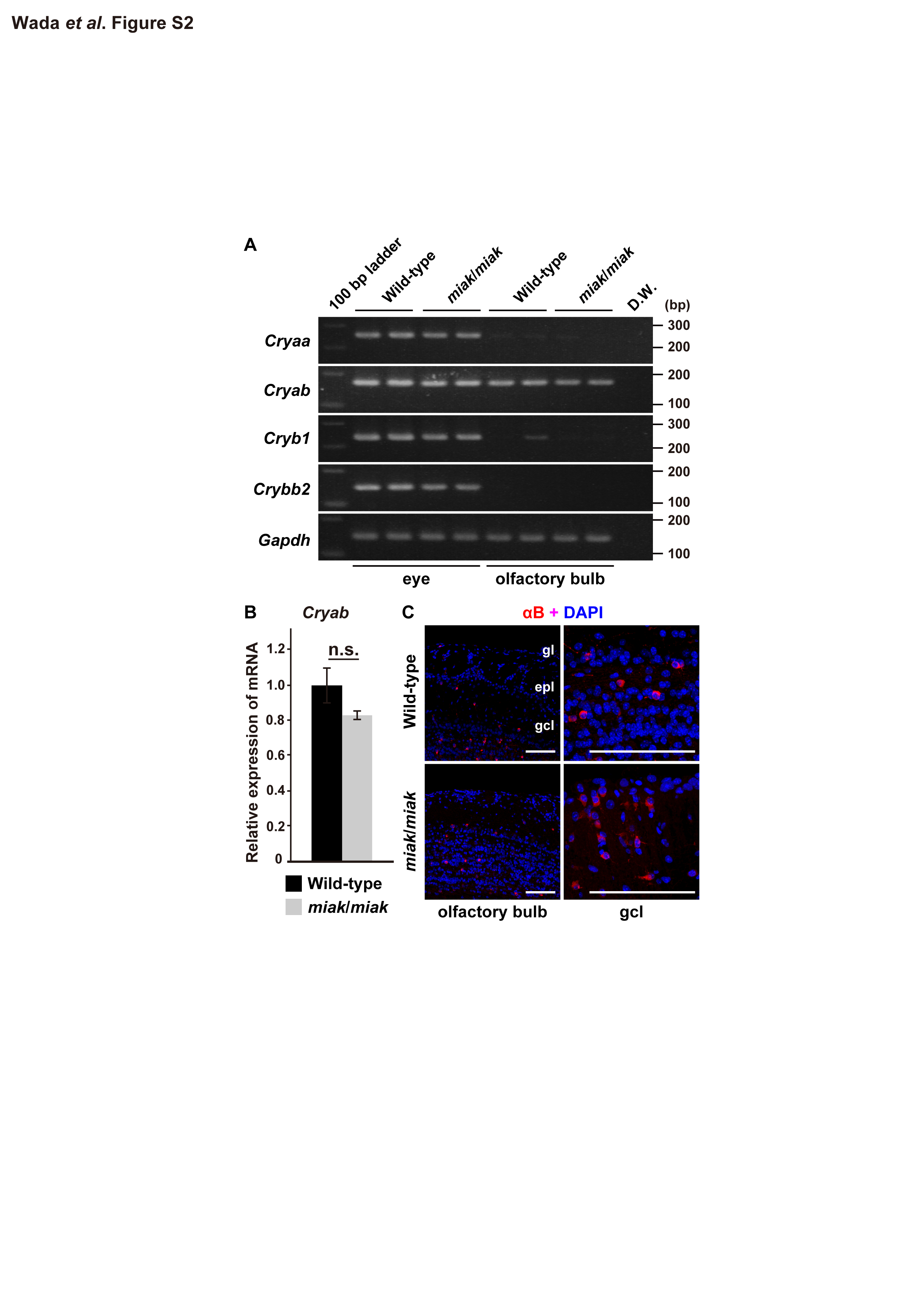

Supplement: Figure S2 — Expression analysis of αA and αB-crystallins in olfactory bulb in wild-type and miak mice. A. Comparison of crystallin (Cryaa, Cryab, Cryb1 and Crybb2) expression between wild-type and miak/miak mice at P30 by RT-PCR. cDNA integrity was confirmed with Gapdh control band (bottom panel). B. Relative expression level of Cryab transcript in olfactory bulb of wild-type and miak/miak at P30. The values shown in each graph indicate the mean relative expression levels and the SDs of triplicates. The expression levels in wild-type olfactory bulbs were assigned an arbitrary value of 1 for comparative purposes. n.s. no significant difference. C, D. Immunohistochemistry of αA-crystallin (C) and αB-crystallin (D) in olfactory bulb of wild-type (top) and miak/miak (bottom) mice at P30. The right panels indicated higher magnified images of periglomerular layer (pgl). gl, glomerular layer; epl, external plexiform layer. Scale bar = 100 µm. (TIF) [file pone.0111432.s002.tif]

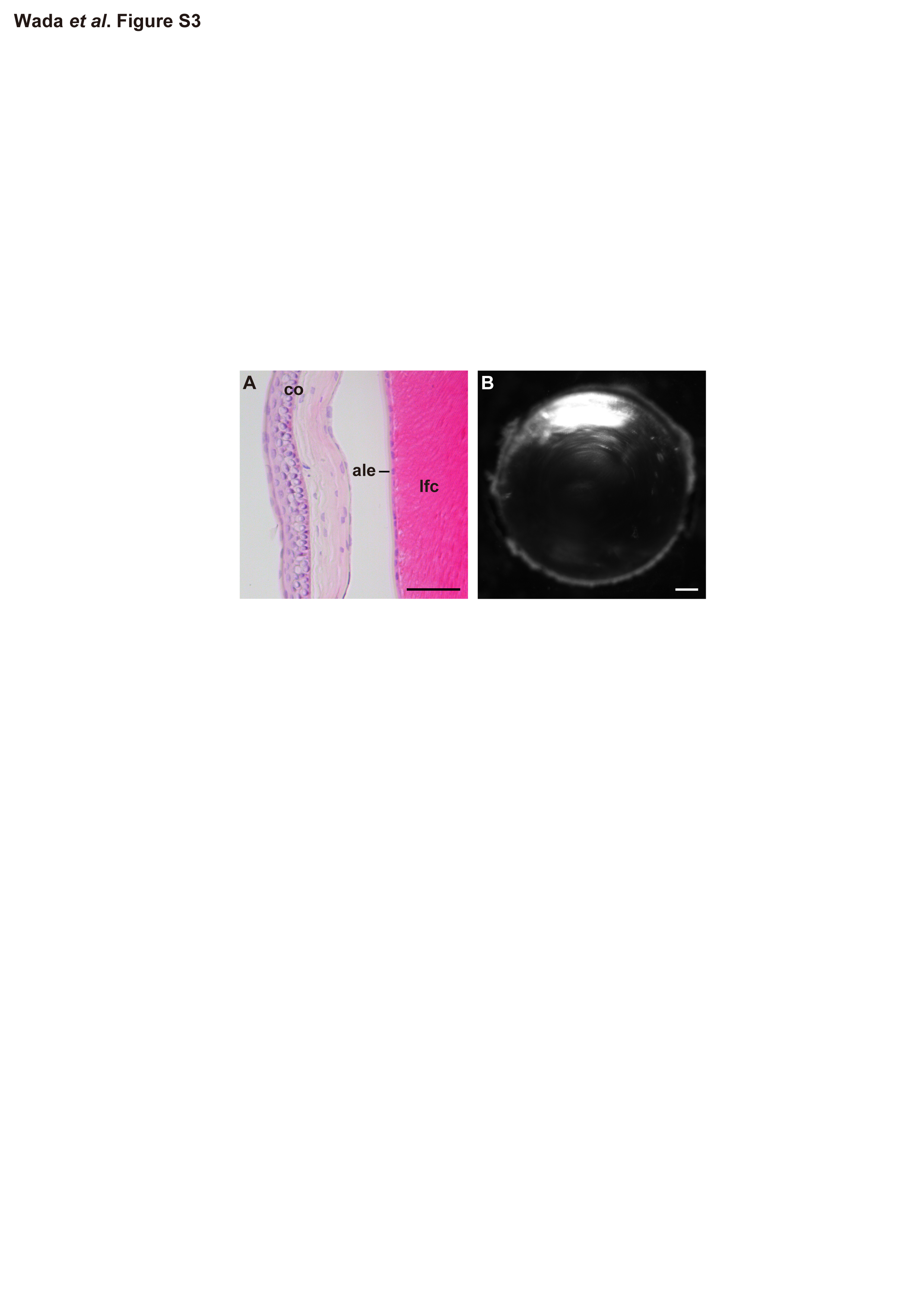

Supplement: Figure S3 — Lens phenotypes in miak /+ heterozygous mice. A. Highly magnified image of the cornea and lens epithelium from the lens section in miak/+ mouse at 6 weeks of age. co, cornea; ale, anterior lens epithelium; lfc, lens fiber cell. Scale bar = 100 µm. B. Dark field imaging of the dissected lens from miak/+ mouse at 10 months of age. The procedure for phenotyping was previously described [11]. The miak/+ mice show normal transparency. Scale bar = 500 µm. (TIF) [file pone.0111432.s003.tif]
